# *Marinobacter* Dominates the Bacterial Community of the *Ostreococcus tauri* Phycosphere in Culture

**DOI:** 10.3389/fmicb.2016.01414

**Published:** 2016-09-07

**Authors:** Josselin Lupette, Raphaël Lami, Marc Krasovec, Nigel Grimsley, Hervé Moreau, Gwenaël Piganeau, Sophie Sanchez-Ferandin

**Affiliations:** ^1^Sorbonne Universités, Université Pierre et Marie Curie Paris 06, UMR 7232 Biologie Intégrative des Organismes Marins, Observatoire OcéanologiqueBanyuls-sur-Mer, France; ^2^Centre National de la Recherche Scientifique, UMR 7232 Biologie Intégrative des Organismes Marins, Observatoire OcéanologiqueBanyuls-sur-Mer, France; ^3^CEA/CNRS/INRA/Université Grenoble Alpes, UMR 5168 Laboratoire Physiologie Cellulaire VégétaleGrenoble, France; ^4^Sorbonne Universités, Université Pierre et Marie Curie Paris 06, USR 3579 Laboratoire de Biodiversité et Biotechnologies Microbiennes, Observatoire OcéanologiqueBanyuls-sur-Mer, France; ^5^Centre National de la Recherche Scientifique, USR 3579 Laboratoire de Biodiversité et Biotechnologies Microbiennes, Observatoire OcéanologiqueBanyuls-sur-Mer, France

**Keywords:** *Ostreococcus tauri*, *Marinobacter* sp., picoalgae, bacteria, interactions, phytoplankton

## Abstract

Microalgal–bacterial interactions are commonly found in marine environments and are well known in diatom cultures maintained in laboratory. These interactions also exert strong effects on bacterial and algal diversity in the oceans. Small green eukaryote algae of the class Mamiellophyceae (Chlorophyta) are ubiquitous and some species, such as *Ostreococcus* spp., are particularly important in Mediterranean coastal lagoons, and are observed as dominant species during phytoplankton blooms in open sea. Despite this, little is known about the diversity of bacteria that might facilitate or hinder *O. tauri* growth. We show, using rDNA 16S sequences, that the bacterial community found in *O. tauri* RCC4221 laboratory cultures is dominated by γ-proteobacteria from the *Marinobacter* genus, regardless of the growth phase of *O. tauri* RCC4221, the photoperiod used, or the nutrient conditions (limited in nitrogen or phosphorous) tested. Several strains of *Marinobacter algicola* were detected, all closely related to strains found in association with taxonomically distinct organisms, particularly with dinoflagellates and coccolithophorids. These sequences were more distantly related to *M. adhaerens, M. aquaeoli* and bacteria usually associated to euglenoids. This is the first time, to our knowledge, that distinct *Marinobacter* strains have been found to be associated with a green alga in culture.

## Introduction

Phytoplankton, together with viruses, bacteria, and micrograzers constitute different communities of species which all play fundamental roles in the functioning of microbial food web (Buchan et al., 2014). Phytoplankton and bacterial communities are closely linked in coastal marine environments (Fuhrman et al., 1980; Rooney-Varga et al., 2005; Amin et al., 2015) and bacterial–algal interactions play a major role in determining bacterial and algal diversity in the ocean (Schäfer et al., 2002). Detailed knowledge of these interactions is thus crucial for understanding marine ecosystems. Phytoplankton exudates can be important substrates for bacteria, especially in early phytoplankton bloom conditions (Fouilland et al., 2013) although other carbon sources might also be important for bacterial growth (Fouilland and Mostajir, 2010). In turn, some bacteria are known to inhibit or promote microalgal growth (Cole, 1982; Fukami et al., 1997; de-Bashan et al., 2004). The volume of water closely surrounding algal cells in which many metabolic exchanges may occur, is called the phycosphere (Bell and Mitchell, 1972). It is analogous to the rhizosphere in soils and it has direct implications for nutrient fluxes to and from algal cells (Amin et al., 2012b). In this niche, bacteria can live freely around microalgae and interact through metabolic fluxes via the environmental medium, or they may be more closely associated with the cells, such as epiphytic or endophytic bacteria. Epiphytic bacteria adhere to the microalgal surface (Bell and Mitchell, 1972; Shapiro et al., 1998) with a tight functional association (Middelboe et al., 1995; Smith et al., 1995; Grossart et al., 2005). Endophytic bacteria are able to develop inside microalgal cells and have been seen in Chlorophyta (Kawafune et al., 2012). Both commensalism and/or competition for micronutrients may occur between phytoplankton and bacteria (Bratbak and Thingstad, 1985; Amin et al., 2012a). Bacteria might positively (stimulation) or negatively (inhibition, alteration of physiology, death) influence phytoplankton dynamics. As examples, stimulation of phytoplankton growth by bacteria can occur via the production of vitamins (Haines and Guillard, 1974; Kurata, 1986; Croft et al., 2005; Kuo and Lin, 2013), siderophores (Martinez et al., 2000, 2003; Amin et al., 2009) or phytohormones (de-Bashan et al., 2008) like auxin (Gonzalez and Bashan, 2000). In contrast, bacteria can even kill the algae by the secretion of algicidal compounds (Mayali and Azam, 2004).

Some studies demonstrated the presence of specific bacterial communities associated with algal blooms in marine environments (Gonzalez et al., 2000; Buchan et al., 2014). In axenic (i.e., exempt of bacteria) microalgal monocultures, microalgal growth may be unstable and prone to perturbation (Kazamia et al., 2014), highlighting again the importance of microalgalbacterial interactions, not only in natural environments but also in laboratory culture conditions. However, except for some species (Alavi et al., 2001; Green et al., 2004, 2015; Jasti et al., 2005), particularly diatoms (Grossart et al., 2005; Sapp et al., 2007a,b; Eigemann et al., 2013; Amin et al., 2015; Mishamandani et al., 2016), the bacterial diversity associated with microalgae species is still poorly known. Small green algae belonging to the order Mamiellales are ubiquitous in the world oceans including the Arctic zone (de Vargas et al., 2015), and are of prime importance in the ecology of Mediterranean coastal lagoons. Among this group, the smallest free-living eukaryotic cell *Ostreococcus tauri* was discovered in the Mediterranean lagoon Thau 20 years ago (Courties et al., 1994), and can be observed as a dominant species during phytoplanktonic blooms in coastal seas (O’Kelly et al., 2003). In such lagoons or coastal regions where limnic and oceanic waters meet, the environment is more variable than in open sea, strongly influencing bacterial diversity (Glöckner et al., 1999; Herlemann et al., 2011; Bižić-Ionescu et al., 2015). *O. tauri* is also being used as a model organism for studying diverse environmental problems like sensitivity to herbicides (Sanchez-Ferandin et al., 2013) or tolerance to polluants like arsenic (Zhang et al., 2013). Despite the wealth of genomic data available for this species (Derelle et al., 2006; Blanc-Mathieu et al., 2014), the bacterial community associated with the *O. tauri* phycosphere is poorly understood, and the nature of the interactions between *O. tauri* and bacteria remains an open question (Abby et al., 2014).

Here, we focus on the nature and dynamics of the microbiome of *O. tauri* RCC4221 across a large range of culture conditions. Surprisingly, we provide evidence that bacteria from one single genus, *Marinobacter* (γ-proteobacteria, order Alteromonadales) is largely predominant across culture conditions.

## Materials and Methods

### *Ostreococcus tauri* RCC4221 Culture in Different Conditions

*Ostreococcus tauri* strain RCC4221 was isolated in 1994 from the North-West Mediterranean Thau lagoon (Courties et al., 1994) and maintained in the laboratory (cultures and cryopreservation). The *O. tauri* strain was grown in liquid medium in aerated flasks (Sarstedt), in growth chambers at 20±1°C and white light at around 100 μmol photons.m^-2^.sec^-1^ using three photoperiods (LD 08:16; LD 12:12; and LD 14:10). Two culture media were prepared, one with natural sea water, L1-MOLA (Guillard and Hargraves, 1993) and one with artificial sea water, F/2-ESAW (Harrison et al., 1980). Both L1-MOLA and F/2-ESAW were produced by adding nitrogen (NaNO_3_), phosphorus (NaH_2_PO_4_) and vitamins (B1, B12, and H) but at different concentrations (**Supplementary Table S1**). Only L1-MOLA was prepared from seawater, collected from 20 m below sea level at the MOLA station and kept several weeks in the dark before use. The seawater was filtered through 0.22 μm and autoclaved. For nutrient limitation experiments, four conditions were tested: L1-MOLA, F/2-ESAW, F/2-ESAW 50%N (half nitrogen concentration), and F/2-ESAW 10%P (one tenth phosphorous concentration) (see **Supplementary Table S1** for the detailed composition of each medium used in this study). For each tested condition, cultures were prepared in triplicates. Considering all of the culture experimentations (three photoperiods and four culture media conditions mentioned above), a total of 12 different conditions were used in triplicate, thus 36 different flasks maintained in incubators. Half a milliliter of each culture was collected daily during 35 days and then fixed 10 min at room temperature in the dark with glutaraldehyde (Sigma #G5882 – final concentration: 1%) before frozen in liquid nitrogen and stored at -80°C.

### Cell Concentrations and Growth Rates

Cell concentrations were determined using flow cytometry (FACSCantoII^TM^, Becton Dickinson, San Jose, CA, USA). *O. tauri* cells were detected using the red fluorescence emission (FL3) of chlorophyll pigments. For enumeration of bacteria, nucleic acids were labeled with SYBR^®^ Green I (Lonza, # 50512) and were detected by green fluorescence (FL1) (Marie et al., 1997). Two kinds of bacteria were distinguished: HNA (High apparent Nucleic Acid content) and LNA (Low apparent Nucleic Acid content) bacteria (Gasol et al., 1999). Growth rates (μ_max_) were determined from cell concentrations measured at different times with the following equation (Levasseur et al., 1993):

(1) $$\begin{array}{c} \;\mu_{\max}\;=\;\ln(N_{2}/N_{1})/(t_{2}\;-\;t_{1}) \end{array}$$

(where *N*_1_, *N*_2_, are cell abundances in the exponential phase at times *t*_1_ and *t*_2_).

### Endpoint Dilutions of *O. tauri* RCC4221 for Isolation of Associated Bacteria

An *O. tauri* RCC4221 culture was diluted serially to permit isolation of single microalgal cells in individual culture wells [by “extinction dilution” or “endpoint dilution,” see for example (Taylor, 1962)], reducing also the density of bacteria. Sixteen independent lines obtained in this way from a single cell were maintained similarly by enumeration and dilution through 27 serial single-cell endpoint dilutions (about 500 cell divisions over the 54 weeks of culture). All of the cell lines were grown in L1 medium in 24 well plates with single-cell endpoint dilutions at every 14-day sub-culturing step (Krasovec et al., 2016) (**Table 1**). Each line was then screened on marine agar plates to isolate bacterial colonies and identify their diversity by PCR. The W18 bacterial forward (5′-GNTACCTTGTTACGACTT-3′) and W02 universal reverse (5′-GAGTTTGATCMTGGCTCAG-3′) primers were used (Godon et al., 1997). PCR were run with the Kapa Extra HS mix (CliniSciences) and standard amplification (35 cycles of 15 s at 95°C, 15 s at 50°C and 1 min 20 s at 72°C). Amplified DNAs were sequenced with Sanger method by Cogenics (Takeley, Essex, UK) and Bio2Mar platform (Banyuls-sur-mer, France).

**Table 1:** Summary of the different experimental conditions.

Photoperiod	Experimental conditions	Number of *O. tauri* cultures	Samples for Sanger sequencing	Samples for Illumina sequencing
08:16	Serial endpoint dilutions cultures	16	48	None
08:16 12:12 14:10	L1-MOLA	Cultures in triplicates	384 for all photoperiods and all experimental conditions	16 photoperiod 14:10 LAT, EXP, STA, DEC for all experimental conditions
	F/2-ESAW			
	F/2-ESAW 50%N			
	F/2-ESAW 10%P			

### Identification of Cultivable and Total Bacteria in *O. tauri* RCC4221 Standard and Limiting Nutrient Conditions

For each tested culture condition, aliquots of 100 μL of *O. tauri* cultures were spread onto L1-MOLA solid medium plates every 5 days. After 2 days of incubation in the same conditions as the ones used for the incubation of cells in liquid medium (see the section of Materials and Methods), DNA samples extracted from four morphologically identical colonies per condition were sequenced for a total of 12 conditions (384 sequences; **Table 1**). For total bacterial diversity identification, an aliquot of 100 μL of culture was taken each day during the growth of *O. tauri* RCC4221, fixed in glutaraldehyde 1% (Sigma), frozen in liquid nitrogen, and stored at -80°C. Then, in order to follow bacterial diversity at specific times along *O. tauri* growth, aliquots were pooled following the four different *O. tauri* RCC4221 growth phases: latency (LAT), exponential (EXP), stationary (STA) and decline (DEC) phases. Only samples corresponding to the LD 14:10 condition (for which the whole *O. tauri* growth from LAT to DEC phases was observed) were extracted for subsequent ribosomal 16S DNA sequencing (**Table 1**).

### Statistical Analyses and Graphical Representations

Statistical analyses were performed with R version 3.2.0 software^1^. *t*-tests were done on paired samples by permutation (t.paired.perm.R function) ^2^ were performed. Graphical views from R and Excel were saved in pdf.

### High Throughput Sequencing Data Analysis

Sixteen different conditions were analyzed, using four samples (corresponding to the four *O. tauri* different growth phases: latent period, exponential growth, stationary phase, decline) for each medium. The samples were named OtL1LAT, OtL1EXP, OtL1STA, and OtL1DEC for L1 culture medium; Otf2LAT, Ot f2EXP, Ot f2STA, and Ot f2DEC for f/2 culture medium; OtPLAT, OtPEXP, OtPSTA, and OtPDEC for F/2 (50% [P]); and OtNLAT, OtNEXP, OtNSTA, and OtNDEC for F/2 (10% [N]). For each condition, DNA extraction was performed by using the modified CTAB method (Winnepenninckx et al., 1993). Total microbial 16S rDNA diversity of each of these 16 DNA samples was estimated by Illumina, 2 × 300 bp PE sequencing 20,000 PCR-amplified sequences (MrDNA, Molecular Research Laboratory, 503 Clovis Road, Shallowater, TX 79363 USA). The 16S rRNA gene V4 variable region PCR primers 515/806 with barcode on the forward primer were used in a 28 cycles PCR using the HotStarTaq Plus Master Mix Kit (Qiagen, USA) under the following conditions: 94°C for 3 min, followed by 28 cycles of 94°C for 30 s, 53°C for 40 s and 72°C for 1 min, after which a final elongation step at 72°C for 5 min was performed. After amplification, PCR products were checked in 2% agarose gel to determine the success of amplification and the relative intensity of bands. Sixteen samples were pooled together in equal proportions based on their molecular weight and DNA concentrations. Pooled samples were purified using calibrated Ampure XP beads. Then the pooled and purified PCR products were used to prepare the Illumina DNA library. Sequencing was performed at MR DNA on a MiSeq following the manufacturer’s guidelines. Sequence data were processed using MR DNA analysis pipeline ^3^ and analyzed using QIIME software (Quantitative Insights Into Microbial Ecology)^4^ (Caporaso et al., 2010). In summary, sequences were joined, depleted of barcodes then sequences <150 bp removed, and sequences with ambiguous base calls removed. A 97% cut-off for sequence identity was used for classification into OTUs. The sequences obtained were dominated by *O. tauri* mitochondrial sequences (>85%) and the analysis was conducted after eliminating these sequences, leaving 97362 bacterial sequences of interest spread among the 16 different conditions (roughly 6085 bacterial sequences per condition). Sequences were submitted to GenBank with the project reference (BioProject ID) PRJNA328274.

### Alignments and Phylogenetic Reconstructions

Among the 97362 total sequences obtained, 88179 (90.6%) were assigned to *Marinobacter* spp., clustered at 97% identity threshold inside six distinct OTUs after eliminating sequencing errors (instead of 799 initially obtained comprising 617 OTUs with one sequence and 176 OTUs with less than 200 sequences). The majority of *Marinobacter* sequences (95.3%) grouped inside one unique OTU (84817 sequences in OTU_7771). Among the remaining sequences, 6% (5821 sequences) were assigned to the *Hyphomonas* genus, and clustered in two main OTUs. The majority of *Hyphomonas* sequences (96.4%) grouped inside one unique OTU (5609 sequences in OTU_6113). The alignment of OTU sequences with annotated NCBI 16S rDNA sequences was performed using Clustal W (Thompson et al., 1994) implemented in MEGA 6.1 software (Tamura et al., 2013). The sequence alignment was then adjusted manually. The best evolutionary model was chosen using MEGA 6.1 software and resulted in Kimura two parameters with a Gamma correction. Two phylogenetic trees were constructed following this model by Neighbor-Joining (NJ) and Maximum Likelihood (ML) approaches using 1000 bootstrap replicates. Gram positive bacteria (*Staphylococcus*) and Gram negative δ-proteobacteria (*Geobacter*) were used as outgroups. Given that the two topologies obtained from the two phylogenetical methods were highly similar, only the NJ phylogenetic tree was shown with the bootstraps values resulting from each method.

## Results

### Abundance and Dynamics of Bacteria in the *O. tauri* Phycosphere

**FIGURE 1:**
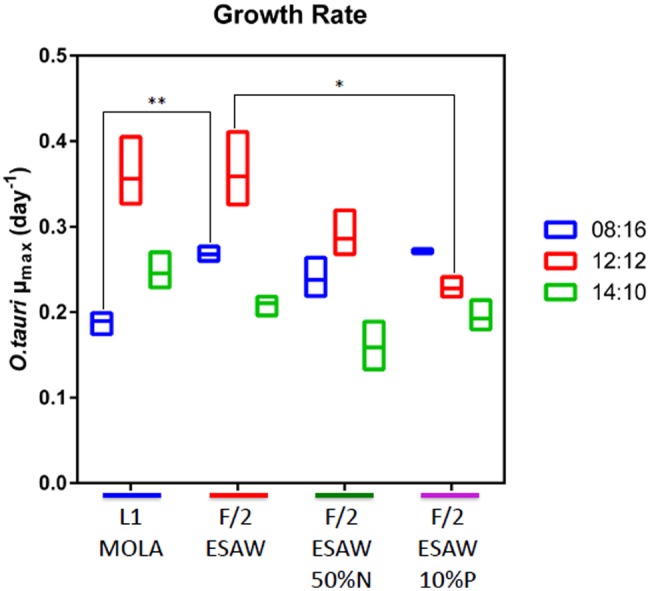
***O. tauri* RCC4221 growth rates comparison in four different media and three different photoperiods.** Media: L1-MOLA, F/2-ESAW (complete media), F/2-ESAW 50%N (nitrogen concentration divided per 2) and F/2-ESAW 10%P (phosphorus concentration divided per 10). Photoperiods: LD 12:12, LD 08:16, LD 14:10. ^∗^*p* < 0.05 and ^∗∗^*p* < 0.005.

**FIGURE 2:**
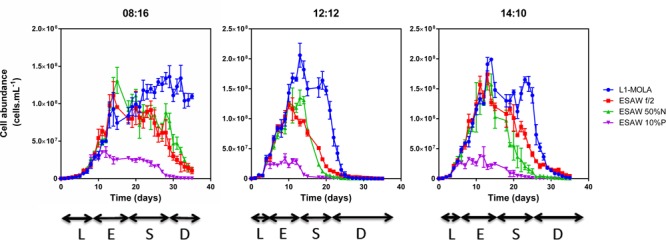
***O. tauri* RCC4221 growth for 35 days in four different media and three different photoperiods.** Media: L1-MOLA, F/2-ESAW (complete medium), F/2-ESAW 50%N (nitrogen concentration divided per 2) and F/2-ESAW 10%P (phosphorous concentration divided per 10). Photoperiods: LD 08:16, LD 12:12, LD 14:10. Growth Phases: L, latency; E, exponential; S, stationary, and D, decline.

**FIGURE 3:**
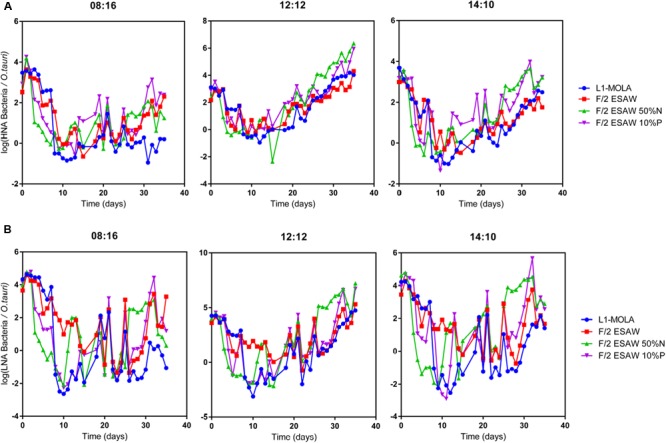
***Ostreococcus tauri* RCC4221-bacterial growth ratios for 35 days in four different media and three different photoperiods.** Media: L1-MOLA, F/2-ESAW (complete), F/2-ESAW 50%N (nitrogen concentration divided per 2) and F/2-ESAW 10%P (phosphorous concentration divided per 10). Photoperiods: LD 08:16, LD 12:12, LD 14:10. **(A)** log (HNA bacteria/*O. tauri*) growth ratio. **(B)** log (LNA bacteria/*O.tauri*) growth ratio.

Globally, the highest *O. tauri* RCC4221 maximal growth rates in exponential phase were observed for cells cultivated in L1-MOLA and F/2-ESAW media at LD 12:12 light cycle condition (**Figure 1**). Interestingly, maximal growth rate appears more important in F/2-ESAW medium than in F/2-ESAW 10%P at this LD 12:12 photoperiod (*p*-value < 0.05) (**Figure 1**). In the other photoperiods, no significant difference was observed between the distinct F/2-ESAW media. We can also observe a decrease in *O. tauri* maximal growth rate in L1-MOLA compared to the one of cells cultivated in F/2-ESAW at LD 08:16 light cycle condition (certainly because of a longer exponential growth phase in L1-MOLA than in F/2-ESAW with minimal light) (*p*-value < 0.005) (**Figure 1**). After 20 days of growth, the highest *O. tauri* cell abundances were observed in L1 MOLA medium whatever the photoperiodic conditions (**Figure 2**). Whatever the culture medium or the photoperiodic condition, different phases of *O. tauri* growth can be identified and are described in **Figure 2**. However, depending upon the different experimental approaches used, these phases can exhibit various durations (**Figure 2**). Globally, the cell concentration reached in the different culture conditions is the lowest in ESAW 10%P whatever the other experimental conditions (**Figure 2**). The relative abundance of total bacteria in *O. tauri* RCC4221 cultures was measured over 35 days in the 12 different conditions (**Figure 3**). HNA (High content DNA) and LNA (Low content DNA) bacteria (Gasol et al., 1999) were observed in the different culture conditions (**Figure 3**). They were considered as dominant when log (HNA bacteria and LNA bacteria/*O. tauri*) ratios >1 (**Figure 3**). Overall, the lowest bacterial abundances were observed when the cultures were grown in L1 MOLA medium (**Figure 3**) and the highest proportions of *O. tauri* cells relatively to bacteria were observed during the exponential growth phase (approximately after 10 days growth) (**Figures 2** and **3**). However, whatever the culture conditions, the highest densities of HNA and LNA bacteria were present in *O. tauri* RCC4221 culture during latency (from 0 to 8 days approximately) and decline (starting from 25 days) phases (**Figures 3A,B**). Since some bacteria may be adhering to algal cells, these figures probably underestimate the actual number of bacteria present. The latency and decline phases are advantageous for the development of LNA bacteria while the exponential phase is more suitable for the development of HNA bacteria (except in F2-ESAW medium). A majority of LNA bacteria [with log (LNA bacteria/*O. tauri*) ratio around 2] are simultaneously present with microalgal cells in exponential phase in F2-ESAW medium (**Figure 3B**). The proportion of LNA bacteria increases when cultivated in F/2-ESAW 50%N and F/2-ESAW 10%P media compared to L1-MOLA medium and are predominant in F/2-ESAW medium whatever the photoperiod condition (**Figure 3B**). In contrast, the development of HNA bacteria was higher in nitrogen-limited medium (except in LD 08:16 photoperiodic condition) and in phosphorus-limited medium in a period corresponding to the stationary phase (from 15 to 25 days approximately) in all tested photoperiodic conditions (**Figure 3A**) while LNA bacteria seem predominant starting from 25 days (**Figure 3B**).

### Diversity of Culturable versus Total Bacteria during *O. tauri* RCC4221 Growth

**FIGURE 4:**
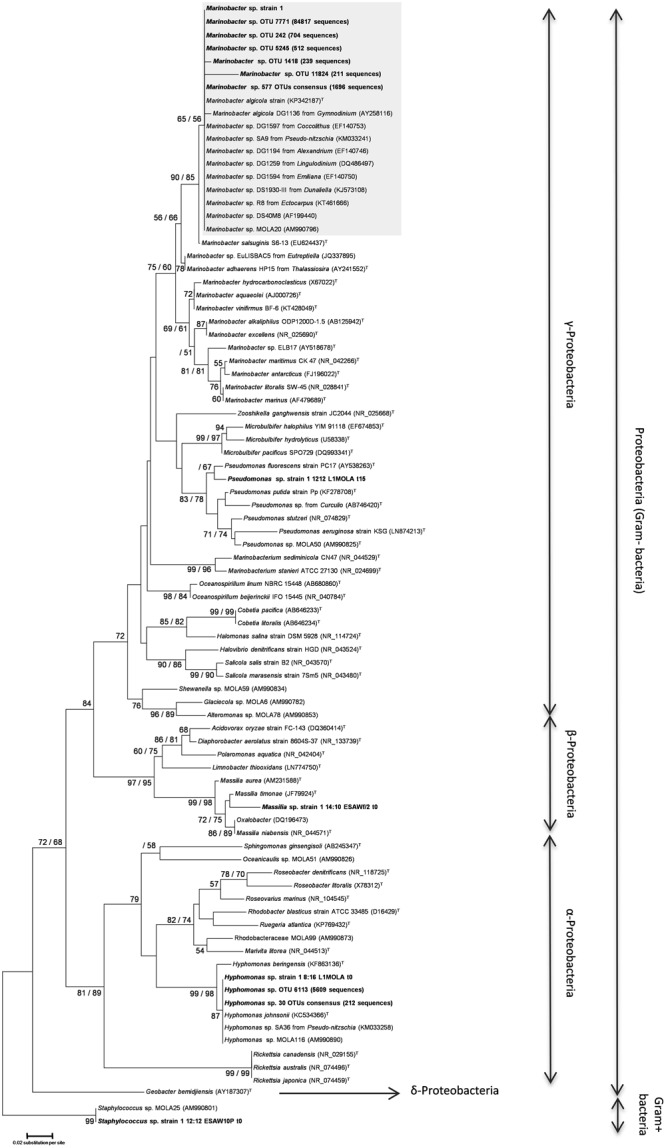
**Neighbor-Joining phylogenetic tree obtained from partial 16S DNA alignment of bacterial sequences obtained from this study and from GenBank.** Numbers reported on the nodes of the tree are the bootstrap values (BP) obtained from NJ and ML methods (BP_NJ_/BP_ML_). Only those superior to 50 are reported. The gray box shows *Marinobacter* sequences affiliated to *M. algicola* species from our phylogenetic reconstructions. The name “*Marinobacter* sp. strain 1” refers to the Sanger-sequencing of *Marinobacter* obtained from the culture-based approach. Sequences obtained in this study were submitted to GenBank with the project reference (BioProject ID) PRJNA328274.

Whatever the culture conditions used in the culture-dependent approach, bacteria from the γ-proteobacteria group were largely dominant. From the 16 *O. tauri* independent lines that were endpoint diluted 27 times through serial subcultures, all the bacteria isolated on either solid Marine Agar or L1 media exhibited one unique 16S sequence (“*Marinobacter* sp. strain 1,” **Figure 4**), closely related to the *Marinobacter* genus (γ-proteobacteria, order Alteromonadales) (**Table 2**; **Figure 4**). The full-length 16S sequence showed 98% identity to the *Marinobacter algicola* and *Marinobacter* sp. DS1930-III sequences. Bacteria isolated on L1 solid medium during the 35 days of *O. tauri* RCC4221 growth in the different culture conditions also exhibited the same full-length 16S sequence with 98% identity to the *Marinobacter algicola* species in 95.3% of the bacteria identified (**Table 2**). Other γ-, β-, and α-culturable proteobacteria were also detected in the different cultures, albeit found much less frequently. One sequence belonging to *Pseudomonas* (γ-proteobacteria) was found with *O. tauri* cultivated in L1-MOLA medium after 15 days-growth under LD 12:12 photoperiod (**Figure 4**); one sequence belonging to *Hyphomonas* (α-proteobacteria) was found with *O. tauri* cultivated in L1-MOLA medium at the beginning of the growth under LD 08:16 photoperiod (**Figure 4**); one sequence belonging to *Massilia* genus (β-proteobacteria) was found with *O. tauri* cultivated in F/2-ESAW medium at the beginning of the growth under LD 14:10 photoperiod (**Figure 4**); and one Gram-positive bacteria sequence, from the *Staphylococcus* genus was found, possibly a contaminant from handling of cultures.

**Table 2:** Numbers of 16S ribosomal RNA sequences analyzed.

Approach	Experimental conditions	Number of bacterial sequences	16S rDNA gene	Percentage of *Marinobacter* sequences	Percentage of *Hyphomonas* sequences
Culture-dependant (solid medium)	Serial endpoint dilutions cultures	48	Complete (1455 bp)	100%	–
	L1-MOLA/F/2-ESAW/F/2-ESAW 50%N/F/2-ESAW 10%P	384	Complete (1455 bp)	95.3%	0.3%
Culture-independent (Illumina)	L1-MOLA/F/2-ESAW/F/2-ESAW 50%N/F/2-ESAW 10%P	97362	Partial (270 bp)	90.6%	6%

*Bacterial colonies were obtained after growth in L1 solid medium. Except for the serial dilution extinction experiment, bacteria were isolated from the 12 different experimental conditions (four different media L1-MOLA, F/2-ESAW, F/2-ESAW 50%N, and F/2-ESAW 10%P- and three different photoperiodic conditions). Bacterial sequences were analyzed after either using Sanger sequencing (culture-dependant approaches) or partial (Illumina) length 16S DNA sequencing (independent of bacterial culture).*

The total bacterial diversity in *O. tauri* RCC4221 cultures was investigated by Illumina sequencing at distinct growth phases (latency, exponential, stationary, and decline phases) during the 35 days’ growth in 14:10 light-dark cycles. Globally, the total diversity observed was entirely congruent with our results using a culture-dependent approach showing a large majority of *Marinobacter* spp. (**Table 2**). More precisely, in spite of the relatively small 16S sequence length available, the total diversity analysis provided evidence for diverse *Marinobacter* strains (**Figure 4**). These partial sequences are clearly closely related to a group comprising free-living *M. salsuginis, M. algicola, M.* MOLA20 and diverse *Marinobacter* spp. found associated with different microalgae (BP_NJ_ = 90, BP_ML_ = 85, **Figure 4**). More precisely, they exhibit a close relationship to the *M. algicola* clade (BP_NJ_ = 65, BP_ML_ = 56, **Figure 4**). In addition to the OTU 7771 (84817 sequences) found as 99% identical to the previously identified culturable *Marinobacter* strain (*Marinobacter* sp. strain 1, **Figure 4**), several variants were found from the total diversity analysis (the following percentages of identity are calculated from the partial sequences used in the final alignment): OTU 242 (704 sequences), that is 96.7% identical to OTU 7771, OTU 5245 (512 sequences), 96% identical to OTU 7771, OTU 1418 (239 sequences), 96.7% identical to OTU 7771, OTU 11824 (211 sequences), 96.7% identical to OTU 7771 (**Figure 4**). All of the *Hyphomonas* sequences obtained from Illumina sequencing were closely related to *H. johnsonii* sequences, with strong bootstrap support (BP_NJ_ = 87) in the *Hyphomonas* clade (BP_NJ_ = 99, BP_ML_ = 98) of α-proteobacteria (**Figure 4**).

**FIGURE 5:**
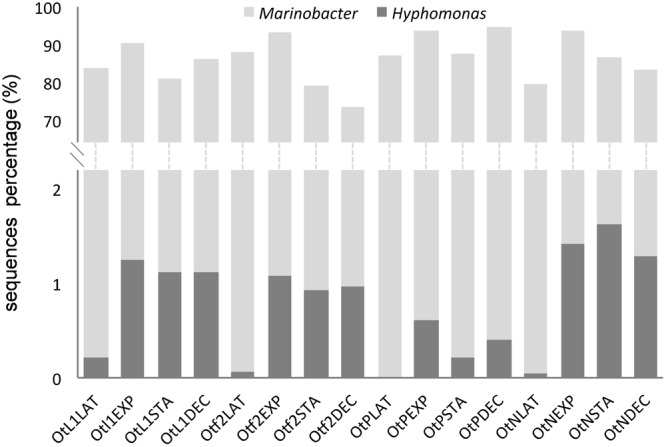
**Proportion of *Marinobacter* and *Hyphomonas* sequences obtained from total bacterial diversity (Illumina) analysis in the four experimental conditions and different *O. tauri* growth phases.** Bacterial sequences were obtained from *O. tauri* RCC4221 cultures in the four different media, noted as OtL1, Otf2, OtN (F/2-ESAW 50%N) and OtP (F/2-ESAW 10%P), and in the four main growth phases (LAT, latency; EXP, exponential; STA, stationary; and DEC, decline). In each culture condition, an increase in the proportion of *Hyphomonas* sequences is observed mainly in the exponential, stationary and decline growth phases.

*Marinobacter* was by far the most frequently found genus and *Marinobacter* sequences were present at all stages and conditions during the growth of *O. tauri* RCC4221 (**Figure 5**). In addition to γ-proteobacteria, sequences from α-proteobacteria (mainly *Hyphomonas* species) were recovered (**Figure 5**) at a low percentage in the culture-dependent approach (0.3%), but at a higher frequency by Illumina sequencing (6%). Whatever the culture conditions, the *Marinobacter* genus always predominated (90.6%, **Table 2**). The highest proportion of *Hyphomonas* sequences was observed during exponential, stationary and decline phases, particularly in L1, F/2 and N-limited media conditions (**Figure 5**).

## Discussion

### *Marinobacter* (γ-Proteobacteria) Was the Most Prevalent Genus Across a Wide Range of Culture Conditions

Globally, the analysis of diversity using Illumina sequencing on total community was entirely congruent with diversity inferred with a culture-dependent approach. Strikingly, all 16 *O. tauri* RCC4221 cultures regularly submitted to serial endpoint dilutions to one single cell per ml contained only sequences from the *Marinobacter* genus. These observations strongly suggest that these bacteria are required for *O. tauri* RCC42221 growth in our culture conditions but we cannot exclude the opportunistic presence of *Marinobacter*, given the enriched cultures media used and the possible use by bacteria of exudates secreted by microalgae, as shown in other bacteria–phytoplankton interactions (Fouilland et al., 2013). Interestingly, in our study, several distinct *Marinobacter* strains were present simultaneously and systematically throughout the growth of the *O. tauri* RCC4221 strain, whatever the conditions tested, as shown by high-throughput Illumina sequencing of PCR-amplified 16S ribosomal gene sequences directly from the algal culture, with no separate bacterial culture step, revealing an unsuspected genetic diversity. This suggests that in general there may be no strict association between one OTU and microalgae, but several closely related OTUs and microalgae, together with *Marinobacter* spp., like those found in association with dinoflagellates, coccolithophorids, and one diatom (Green et al., 2004, 2015; Amin et al., 2009, 2015). To our knowledge, this is the first report of an association between *Marinobacter* strains and a green marine microalga from the class Mamiellophyceae.

*Marinobacter* is the most diversified genus in the Alteromonadaceae (Gauthier et al., 1992; De La Haba et al., 2011) and is commonly found in the oceans worldwide. These bacteria are bacilli with gram-negative walls, halotolerant, with an aerobic metabolism (Gauthier et al., 1992). They use different hydrocarbon sources and are able to perform direct reduction from nitrate to ammonium (known as dissimilatory nitrate reduction) (De La Haba et al., 2011). In our study, the *Marinobacter* sequences obtained from solid culture medium were closely related to sequences from *Marinobacter* sp. MOLA20 (AM990796), a bacterial strain isolated from the Gulf of Lion, and to *Marinobacter* sp. DS1930-III, a bacterial strain which seems to occur as a microbial symbiotic community together with *Halomonas* sp. and *Pelagibaca* sp., and in monocultures of the chlorophyte *Dunaliella salina* (C. Baggesen, unpublished). Several studies have recently described *Marinobacter* strains related to *M. algicola* species in association with microalgal cultures maintained in laboratory and belonging to a wide range of species diversity across the eukaryotic tree of life (Amin et al., 2009; Kuo and Lin, 2013; Le Chevanton et al., 2013; Green et al., 2015). Indeed, among the seven OTUs identified from this study and *Marinobacter* 16S sequences associated with very taxonomically distinct microalgae in the literature (eight OTUs), 14 out of these 15 OTUs cluster all together within the same clade. Members of the genus *Marinobacter* have been detected in numerous dinoflagellate and coccolithophorid cultures (Alavi et al., 2001; Hold et al., 2001; Amin et al., 2009) which all emerged in *M. algicola* clade in our study (**Figure 4**). In addition, a recent analysis of the ectobiotic bacterial diversity associated with the euglenoid *Eutreptiella* sp. revealed the occurrence of abundant γ-proteobacteria, specifically *Marinobacter* (Kuo and Lin, 2013). All of these *Marinobacter* strains were closely related to *M. adhaerens* (Kuo and Lin, 2013). We also included two sequences of *Marinobacter* found associated with different diatoms (one with *Pseudo-nitzschia*, the other with *Thalassiosira*) (Grossart et al., 2004; Amin et al., 2015). Interestingly, the two sequences emerged in different clades, the first one with *M. algicola* and our sequences, the second one with *M. adhaerens* (**Figure 4**).

The presence of *Marinobacter* species in laboratory cultures raises questions about the possibility of a laboratory artifact. Recent findings argue against this, because *Marinobacter* species were also recently found in coccolithophorids and dinoflagellates cultures (Green et al., 2015), although at a much lower proportion in diatom cultures cultivated in the same media (Amin et al., 2009). Rather, we have selected the bacterial species that best supports rapid host growth from a diversity of bacteria observed in the *O. tauri* culture originally isolated (Abby et al., 2014). If *Marinobacter* tends to show a specific adaptation to coccolithophores and dinoflagellates (Green et al., 2010), it appears that, from our study, it is not limited to these groups and can also be extended to Chlorophyta, another distant lineage.

### Other γ-, β-, and α-Proteobacteria Found in *O. tauri* Cultures

Contrary to expectations, we did not detect any *Roseobacter* species although this is the most frequently observed genus in data from surface communities (Morris et al., 2002). However, from both the culture-dependent and Illumina approaches, sequences from the α-proteobacteria *Hyphomonas* sp. were obtained. These sequences are closely related to *H. johnsonii* and to a sequence from a bacterial strain isolated locally (*Hyphomonas* MOLA116, AM990890). However, we exclude the hypothesis that this bacterial strain was introduced as a contaminant in L1 MOLA medium since this medium was autoclaved and no trace of bacteria was detectable by flow cytometry before use. Interestingly, in a recent study, not only *Marinobacter*, but also *Hyphomonas* bacteria were isolated from different isolates of the coastal diatom *Pseudo-nitzschia multiseries* (Amin et al., 2015) and showed the same phylogenetic position that our sequences in our reconstructions. One sequence belonging to *Massilia* genus (β-proteobacteria), particularly rare in marine environments, was also obtained. Gram-positive bacteria were also frequently found in marine microalgal cultures in laboratory, but were probably the result of contamination from handling rather than from the marine environment (Nicolas et al., 2004).

Flavobacteria 16S rDNA sequences were not found from the solid culture medium approach and quasi-absent (0.25% of OTUs) from the Illumina sequencing analysis. Together with α- and γ-proteobacteria (Morris et al., 2002; Sunagawa et al., 2015), they are the bacteria the most commonly detected in microalgal cultures and phytoplanktonic blooms (Buchan et al., 2014). Almost all culturable and visually distinct bacteria isolated from *Chlorella pyrenoidosa, Scenedesmus obliquus, Isochrysis* sp., and *Nitzschia microcephala* microalgal cultures maintained for several years in laboratory belong to the Rhodobacteraceae, Rhizobiaceae, and Erythrobacteraceae families (Schwenk et al., 2014), here again found at very low frequency in *O. tauri* RCC 4221 cultures (0.23, 0.34, and 0.04% of OTUs, respectively).

### Dynamics of Bacterial Growth Depends upon the *O. tauri* RCC4221 Growth Medium

In the light of results from high throughput Illumina sequencing, the proportion of γ-proteobacteria and particularly of *Marinobacter* genus sequences is the largest and encompasses almost all of the bacterial diversity. These different *Marinobacter* strains were subsequently found by flow cytometry as HNA (high apparent nucleic acid content) or LNA (low apparent nucleic acid content) during algal growth. Classically, HNA bacteria are considered as the most active members of a given community while LNA are regarded as inactive, dead or dormant cells (Gasol et al., 1999; Lebaron et al., 2001, 2002). In addition, in numerous studies, both HNA and LNA are considered to be members of different (Zubkov et al., 2001; Fuchs et al., 2005; Mary et al., 2006) or identical (Flaten et al., 2003; Servais et al., 2003) phylotypes (Andrade et al., 2007). Our dominant bacterial population (i.e., *Marinobacter* spp.) was found in both HNA and LNA populations but only further experiments using accurate cell sorting and precise identification of these sorted bacteria would confirm that HNA and LNA have identical ribotypes (David Pecqueur, personal communication). The highest *O. tauri* cell abundance relatively to bacteria was observed during exponential phases where maximal *O. tauri* growth rates were observed. In contrast, the highest bacterial abundance was observed in other phases of the *O. tauri* growth. One interesting fact which retained our attention is the relatively low abundance of *O. tauri* all along the duration of experiments when cultivated in depleted media such as in F/2-ESAW 10%P medium, demonstrating the importance of phosphorous. By the way, HNA bacteria seem particularly abundant in this particular condition. Globally, *O. tauri* cells growth is higher in L1-MOLA medium than in F/2-ESAW one, and appears highly favored compared to both HNA and LNA bacteria and particularly LNA bacteria growths in this medium. One possible explanation is that additional substances present in natural seawater might promote algal growth. Concerning the modalities of *O. tauri* growth, our results tend to show the importance of phosphorous, while nitrogen limitation doesn’t significantly reduce microalgal growth. However, excepted in LD 08:16 photoperiodic condition, there is no clear difference in *O. tauri* RCC4221 maximal growth rates in exponential phase for cells cultivated in L1-MOLA and F/2-ESAW media. We also observed that the bacterial abundance profiles varied with the microalgal culture phases, a higher abundance of bacteria being found in the LAT and DEC phases of *O. tauri* growth. Some bacteria, in particular γ-proteobacteria very often live epiphytically on detritic organic particles (DeLong et al., 1993), perhaps explaining the abundance of bacteria when microalgal growth declines.

### Possible Roles of Bacteria in Algal Cultures

The bacteria isolated in this study have probably survived over several years in continuous algal cultures, where only those bacteria capable of growing under nutrient-rich conditions will survive after successive transfers. Some bacterial groups are able to grow rapidly, out-competing other bacteria in enrichment cultures, as seen in *Marinobacter* species (Handley et al., 2010), when there may be a surplus of organic carbon, or under aerobic to anaerobic conditions (Edwards et al., 2003; Handley et al., 2013). The lifestyle types exhibited by *Marinobacter* strains such as *M. aquaeolei* have been described as opportunistic, being able to utilize urea and phosphonate as alternative of N and P sources, or generalist like *Shewanella, Pseudomonas, Vibrio*, and *Roseobacter* (Singer et al., 2011).

In serial endpoint dilution subcultures, only the bacteria associated with the single algal cells used to establish the culture have the opportunity to be present as the culture is grown and transferred. We showed that *Marinobacter* is present from the beginning of the culture growth. It is almost certain that these bacteria benefit from their coexistence with microalga. Macronutrient concentrations found in algal cultivation media such as F/2-ESAW are about 100-fold higher than those found in coastal marine environments, such as the Bay of Fundy (Guillard and Ryther, 1962; Martin et al., 2001), also favoring bacterial development. Inversely, *O. tauri* RCC4221 cells probably benefit from the presence of bacteria. Indeed, completely axenic cultures of *O. tauri* RCC4221 have not so far been possible to maintain, despite the use of antibiotics treatment protocols. Abby et al. (2014) focused on algal cultures maintained without antibiotics for several years (Abby et al., 2014), and found a total of 1425 *Marinobacter* sequences in an *O. tauri* RCC4221 culture among over 1400000 sequences (thus, representing 0.10%). As in the present study, these sequences were all closely related to *M. algicola*. It is not surprising that Abby et al. (2014) found a greater diversity of different bacterial species present, because cultures were then routinely maintained by subculturing using a larger volume (usually 50 μl) of culture, permitting transfer of a population of bacteria, rather than by dilution through one-cell endpoint dilutions. Over half of all microalgal species require an exogenous supply of vitamin B12 (cobalamin). Among the four B12-dependent enzymes, only one, the methionine synthase, is present in *O. tauri* genome, and *O. tauri* was shown to be auxotrophic for vitamin B12 (Helliwell et al., 2011). In addition, a recent study confirmed *Ostreococcus* to be a thiamine (vitamin B1) auxotroph in laboratory experiments using culture media also containing vitamin B12 (cobalamin) and B7 biotin (Paerl et al., 2015). In this latter study, growth of *Ostreococcus* was limited following serial subcultures in medium lacking B1. To our knowledge, no such experiment has been conducted to explore the effect of vitamin B7 limitation on *O. tauri* growth. Bacteria secreting vitamins can stimulate microalgal growth as vitamin-dependent axenic microalgae cannot survive in nutrient-limited medium (Haines and Guillard, 1974; Kurata, 1986; Croft et al., 2005; Grant et al., 2014). Eighteen clones exhibiting a *Marinobacter* 16S sequence closely related to *M. adhaerens* were obtained from ectobiotic bacteria living with the euglenoid *Eutrepsiella* sp. (Kuo and Lin, 2013). These bacteria provided vitamin B12 and other growth-enhancing factors for the euglenoid (Kuo and Lin, 2013). However, the growth rate of the diatom *Pseudo-nitzschia multiseries* was unaffected when co-cultured with *Marinobacter* in specific experiments where the diatom was previously treated with antibiotics (Amin et al., 2015).

Phytoplankton needs iron in large amounts to support the photosynthetic fixation of carbon. Bacteria from *Marinobacter* genus can produce siderophores (Vraspir and Butler, 2009) and contribute to iron chelation and internalization (Martinez et al., 2000, 2003). Siderophore (like vibrioferrin) production may be a useful chemotaxonomic marker for algal-associated *Marinobacter* species (Amin et al., 2009). Our phylogenetic reconstruction (**Figure 4**) clearly shows the close relationship between our *Marinobacter* strains and the *Marinobacter* sp. DG1194 (isolated from an *Alexandrium* culture), DG1594 (isolated from an *Emiliana* culture) and DG1597 (isolated from a *Coccolithus* culture), all shown to produce and uptake vibrioferrin (Amin et al., 2009) (BP_NJ_ = 65, BP_ML_ = 56, **Figure 4**). In their study, the authors suggested that bacteria may promote algal assimilation of iron (Amin et al., 2009). Bacteria of the *Marinobacter* genus also seem to stimulate the growth of the dinoflagellate *Gymnodinium catenatum* (Bolch et al., 2011), and the accumulation of lipids in the commercially important microalgae *Nannochloropsis* (David Green, personal communication). Although *O. tauri* can be cultured easily in the laboratory, the complete eradication of bacteria is difficult and seems to prevent microalgal growth (unpublished results). This persistence of bacteria in *O. tauri* cultures raises questions regarding the nature of the potential interactions between these microbial populations. Further experiments are needed to investigate the physiological impacts of these newly identified *Marinobacter* strains in our *O. tauri* RCC4221 system.

### Bacteria Associated with Microalgae in Natural Environments

Buchan et al. (2014) monitored the succession of bacterial communities associated with diatoms, dinoflagellates, nanoflagellates, and picophytoplankton blooms in a large-scale study (Buchan et al., 2014). The main bloom-associated bacterial groups were, in order of abundance, α-proteobacteria, Flavobacteriia, and γ-proteobacteria. In the earliest stages of a bloom, phytoplankton release amino acids, organic acids, carbohydrates, and sugar alcohols which are hypothesized to function as chemoattractants for beneficial bacteria, including bacteria that produce phytoplankton growth-promoting compounds, such as vitamins. At the height of the bloom, in response to nutrient-limiting conditions, the release of small molecules by living phytoplankton increases and further stimulates heterotrophic bacterial activity (Buchan et al., 2014). Then, during the waning stage of the bloom, phytoplankton release higher molecular weight macromolecules (polysaccharides, proteins, nucleic acids, lipids, material resulting from cell lysis) (Buchan et al., 2014). In our experiments, we observed a higher proportion of bacteria in the latency (LAT) and decline (DEC) phases of *O. tauri* growth. Some bacteria, in particular γ-proteobacteria very often live as epiphytes on detritic organic particles (DeLong et al., 1993). This phenomenon could explain the abundance of bacteria particularly when the microalgal growth declines. We also observed a higher bacterial diversity (including mainly *Marinobacter*, but also *Hyphomonas* to a lesser extent) during the exponential (EXP), stationary (STA) and decline (DEC) phases of the *O. tauri* RCC4221 growth, which is congruent with the stimulation of heterotrophic bacterial activity observed by Buchan et al. (2014). Lastly, we have to keep in mind that the type of a given algal-bacterial interaction can also change depending on environmental conditions. In laboratory conditions, the interaction between *Scenedesmus obliquus* and a non-identified bacterial community evolves from a mutualistic to a competitive interaction in phosphorus-limited conditions (Danger et al., 2007). A mutualistic phase and a pathogenic phase (where bacteria probably killed dinoflagellate cells), were also observed in co-cultures of *Dinoroseobacter shibae* and *Prorocentrum minimum* (Wang et al., 2014). These observations highlight the need of further experimentation in co-cultures to assess the complexity of bacterial–algal interactions.

## Conclusion

We show that bacterial communities associated with *O. tauri* RCC4221 laboratory cultures are almost exclusively limited to γ-proteobacterial strains from the *Marinobacter* genus, regardless of the growth phases of *O. tauri* RCC4221 or cultures conditions tested (photoperiod and nutrient depletion). Interestingly, *Marinobacter* bacteria identified in *O. tauri* RCC4221 cultures by both culture-dependant and high-throughput sequencing are all closely related to the *M. algicola* clade, regrouping strains known to produce siderophores. Further experiments combining co-cultures with vitamins or nutrient-limiting conditions should provide additional insights in these green algal-bacterial interactions.

## Author Contributions

JL performed experimental approaches, figures, and participated to the writing of the manuscript. RL analyzed high throughput Illumina raw data and participated to the writing of the manuscript. MK performed endpoint dilution experiments and participated to the writing of the manuscript. NG, HM, and GP helped to design the experiments and actively participated to the writing of the manuscript. SS-F conceived the study, participated to the experiments, performed phylogenetic analyses, and wrote the manuscript.

## Conflict of Interest Statement

The authors declare that the research was conducted in the absence of any commercial or financial relationships that could be construed as a potential conflict of interest.

## Footnotes

1 http://www.R-project.org

2 http://adn.biol.umontreal.ca/∼numericalecology/Rcode/

3 www.mrdnalab.com

4 http://qiime.org/

## Supplementary Material

Table S1 **Composition of L1-MOLA (Guillard and Hargraves, 1993) and F/2-ESAW (Harrison et al., 1980) media. (nd, not determined)**.
