# Supplementary material for: Marinobacter Dominates the Bacterial Community of the Ostreococcus tauri Phycosphere in Culture
Source: Front Microbiol. 2016 Sep 7;7:1414. doi: 10.3389/fmicb.2016.01414 (PMC5013054; doi:10.3389/fmicb.2016.01414)
Supplement: Table S1 — Composition of L1-MOLA (Guillard and Hargraves, 1993) and F/2-ESAW (Harrison et al., 1980) media. (nd, not determined). [file Table_1.DOC]

| Final molar concentration (mol/L) | | |
| --- | --- | --- |
| Composition | L1-MOLA medium | F/2-ESAW medium |
| MOLA 20 m-depth | MOLA water | none |
| NaCl | nd | 3.63 x 10^-1^ |
| Na_2_SO_4_ | nd | 2.5 x 10^-2^ |
| KCl | nd | 8.03 x 10^-3^ |
| NaHCO_3_ | nd | 2.07 x 10^-3^ |
| KBr | nd | 7.25 x 10^-4^ |
| H_3_BO_3_ | nd | 3.72 x 10^-4^ |
| NaF | nd | 6.67 x 10^-5^ |
| MgCl_2_ 6H_2_O | nd | 4.71 x 10^-2^ |
| CaCl_2_ 2H_2_O | nd | 9.14 x 10^-3^ |
| SrCl_2_ 6H_2_O | nd | 8.18 x 10^-5^ |
| Major nutriments | | |
| NaNO_3_ | 8.82 x 10^-4^ | 8.8 x 10^-5^ |
| NaH_2_PO_4_· H_2_O | 3.62 x 10^-5^ | 3.62 x 10^-5^ |
| Vitamins solution | | |
| thiamine · HCl (vit. B1) | 2.96 x 10^-7^ | 2.96 x 10^-7^ |
| biotin (vit. H) | 2.05 x 10^-9^ | 4.09 x 10^-9^ |
| cyanocobalamin (vit. B12) | 3.69 x 10^-10^ | 1.48 x 10^-9^ |
| Trace elements solution | | |
| Na_2_EDTA · 2H_2_O | 1.17 x 10^-5^ | 6.56 x 10^-6^ |
| FeCl_3_ · 6H_2_O | 1.17 x 10^-5^ | 6.55 x 10^-6^ |
| MnCl_2_·4H_2_O | 9.09 x 10^-7^ | none |
| ZnSO_4_ · 7H_2_O | 8.00 x 10^-8^ | 2.54 x 10^-7^ |
| CoCl_2_ · 6H_2_O | 5.00 x 10^-8^ | 5.69 x 10^-8^ |
| CuSO_4_ · 5H_2_O | 1.00 x 10^-8^ | none |
| Na_2_MoO_4_ · 2H_2_O | 8.22 x 10^-8^ | 6.12 x 10^-9^ |
| H_2_SeO_3_ | 1.00 x 10^-8^ | none |
| NiSO_4_ · 6H_2_O | 1.00 x 10^-8^ | none |
| Na_3_VO_4_ | 1.00 x 10^-8^ | none |
| K_2_CrO_4_ | 1.00 x 10^-8^ | none |
| CoSO_4_.7H_2_O | nd | 5.69 x 10^-8^ |
| MnSO_4_.4H_2_O | nd | 2.42 x 10^-6^ |
| Na_2_SeO_3_ | nd | 1.00 x 10^-9^ |
| NiCl_2_. 6H_2_O | nd | 6.27 x 10^-9^ |
